# Influence of the patients’ sex, type of dental prosthesis and antagonist on residual bone resorption at the level of the premaxilla

**DOI:** 10.4317/medoral.17079

**Published:** 2011-12-06

**Authors:** María Andrés-Veiga, Cristina Barona-Dorado, María-José-Sandra Martínez-González, Juan López-Quiles-Martínez, José-María Martínez-González

**Affiliations:** 1 Master’s degree in periodontics, oral surgery and dental implants; 2 Associate Professor of Oral Surgery at the Complutense University of Madrid. Assistant Director of the Master’s in Oral and Dental Implant Surgery program, University Hospital of Madrid; 3 Associate Professor of the Master’s in Oral and Dental Implant Surgery program, University Hospital of Madrid; 4 Tenured Full-Time Professor at the Complutense University of Madrid; 5 Full Professor of Maxillofacial Surgery at the Complutense University of Madrid. Head of the Department of Oral and Dental Implant Surgery at the University Hospital of Madrid

## Abstract

Objectives: To analyze the height and width of the ridge at the level of the premaxilla in edentulous patients, evaluating whether the sex of the patient, type of prosthetic rehabilitation and antagonist have an influence. Material and Method: We randomly selected a total of 89 patients, having an average age of 66.21 years old. A total of 308 measurements were made, all of them at the level of the premaxilla, in the intercanine area. As dependent variables, we analyzed the patients’ sex, age and the antagonist: removable (dental) prostheses (RP), fixed (dental) prostheses (FD), natural dentition (ND). As independent variables, we measured the height and residual width in sagittal sections provided by tomographic studies using Dentascan®. Results: We observed a significantly smaller ridge in women versus in men, and in patients whose antagonist was a fixed prosthesis; whereas for the type of prosthesis, we did not observe significant differences between the two categories analyzed. Conclusions: Bone resorption at the level of the premaxilla is a variable process in which a smaller size is observed (height and width) in women and when the antagonist is a fixed prosthesis.

** Key words:** Bone resorption, premaxilla, dental prosthesis, edentulous.

## Introduction

The resorption of the alveolar ridge after dental extraction is a chronic, cummulative and variable process, in which the frequency varies between two patients or even for the same individual (1,2). This circumstance is due to a series of factors that Devlin et al. (3) have categorized as systemic and local factors. The systemic factors include: a decrease in the absorption of calcium, systemic alterations such as osteoporosis, hyperthyroidism, hyperparathyroidism or diabetes, and certain medications such as corticoids or thyroxin, whose prolonged use constitutes risk factors for the onset of osteoporosis (2,4). On the other hand, the local factors include: the status of the alveolar process following the dental extraction (morphology, height and quality of the ridge), cause and type of dental extraction, extension and location of the tooth lost, duration of edentulism, stress on the ridge, parafunctions, antagonist and mucosa-supported prostheses. In addition to these local and systemic factors, the majority of the authors establish the age and sex of the patient as important factors in the resorption of the residual alveolar ridge (5,6).

The purpose of this study was to analyze the size, measured as the height and width, of the ridge at the level of the premaxilla, correlating it with the patient’s sex, type of dental prosthetic rehabilitation and its antagonist. 

## Material and Methods

In order to perform this study, we carried out a retrospective observational study on a sample of 89 patients, with an average age of 56.21 years old. The following inclusion criteria were established: missing teeth at the level of the premaxilla; and as exclusion criteria: previous surgical treatment, cyst or tumor pathology and/or existence of embedded teeth at the level of the premaxilla. The measurements were made at the level of the premaxilla, one for each missing tooth, coming up with a total of 308 missing teeth.

The independent variables studied were: the “sex” and “type of dental prosthesis”, establishing three categories: removable dental prosthesis (RP), fixed dental prosthesis (FP), no type of dental prosthesis (NP) and “antagonist” (natural dentition, removable dental prosthesis, fixed dental prosthesis).

**Figure 1:**
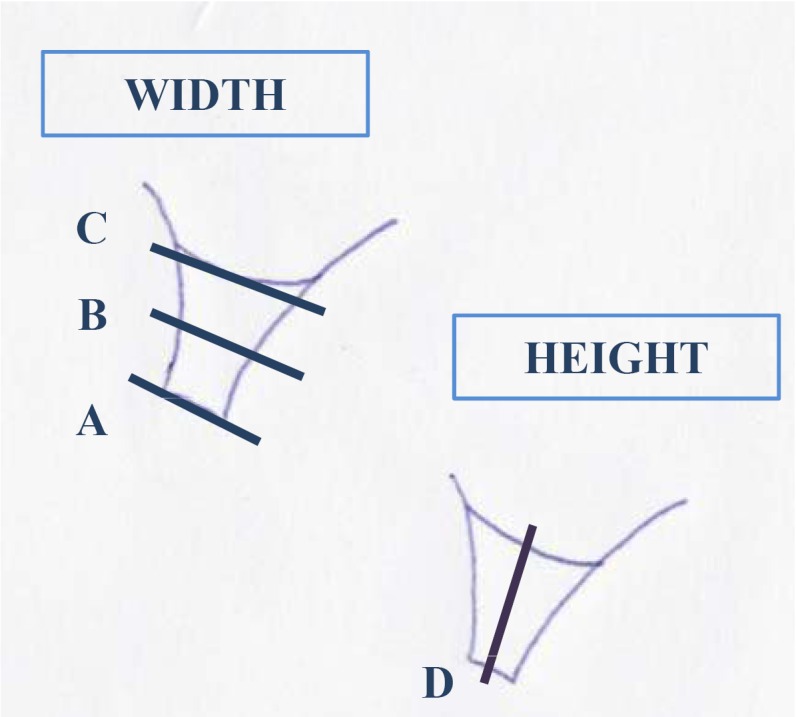
Points of measurement of height and width.

The dependant variables analyzed were the height and width, measured in 4 points: A (width at the coronal level), B (width in the center), C (width at the apical level) and D (height) (Fig. 1).

The statistical analysis of the data was performed by means of an ANOVA test, and for variables with more than 2 categories found to have statistical significance (p<0.05), the Duncan Test was performed afterwards.

## Results

The first variable analyzed was the patients’ sex: of a total of 308 missing teeth, 43.15% (134) were men, whereas 56.49% (174) were women. The results obtained show, through the ANOVA Test, that the height (D) and the width (A, B, C) are less in females versus in males (p<0.01), observing the difference to be statistically significant ( Table 1).

**Figure 2:**
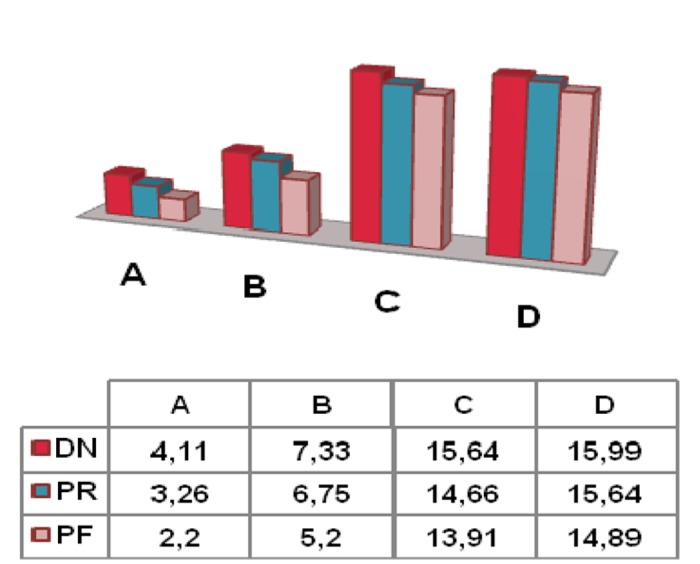
Comparison of height and width according to antagonist: Natural Dentition (ND), Removable Prosthesis (RP), Fixed Prosthesis (FP).

The second variable was the type of prosthesis used on the edentulous premaxilla. Of a total of 308 missing teeth, 3 corresponding to NP were disregarded given that the sample size was not significant, such that the sample was reduced to 305 missing teeth, 88.52% (n: 270) of which corresponded to rehabilitation with RP and 11.43% (n: 35) corresponded to rehabilitation with FP. The results showed that the height (D) and the width at the coronal level (A) and apical level (C) were less in RP (Fig. 2), whereas the width in the center (C) was less in the FP (Fig.2), although these differences were not statistically significant (ANOVA Test) in any of the points measured (p>0.05) ( Table 2).

In the análisis of the size of the residual ridge in terms of the antagonist, we only selected 270 missing teeth in wearers of RP, and 3 categories were established: natural dentition (ND), removable dental prosthesis (RP) and fixed dental prosthesis (FP); which is distributed as follows: 53% (n: 142) of the antagonists corresponded to ND, 35% (n: 94) corresponded to RP, and finally, 12% (n: 34) were FP. As for the results, both in the height (D) as well as in the width (A, B, C), we observed the same pattern, obtaining the lowest values when the antagonist is a FP, whereas the highest values corresponded to RP. The statistical analysis of this variable by means of a Duncan Test revealed statistically significant differences (p<0.01) between FP and the other two categories in the width at the coronal level, central and apical levels, but not in the height (D).

**Table 1:**
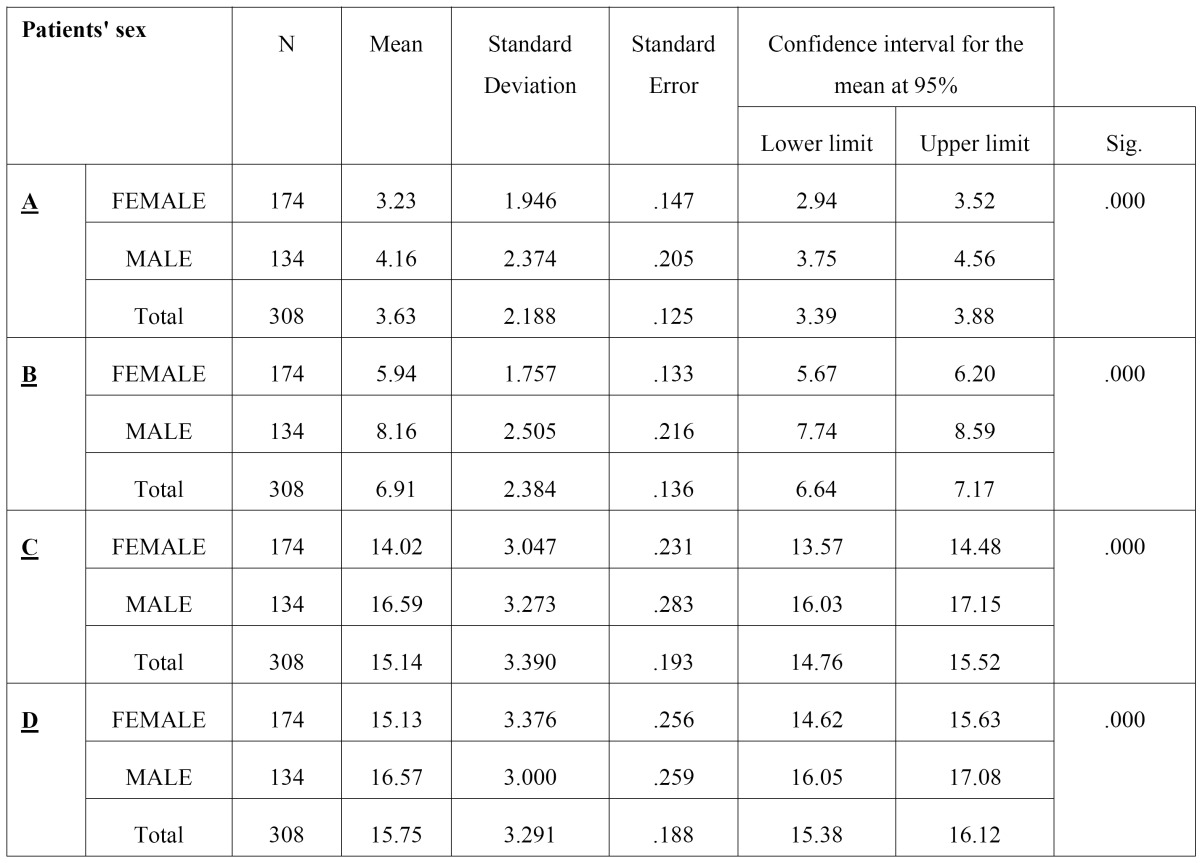
Descriptive and analytical analysis of the variable “Patients’ sex” (ANOVA Test).

**Table 2:**
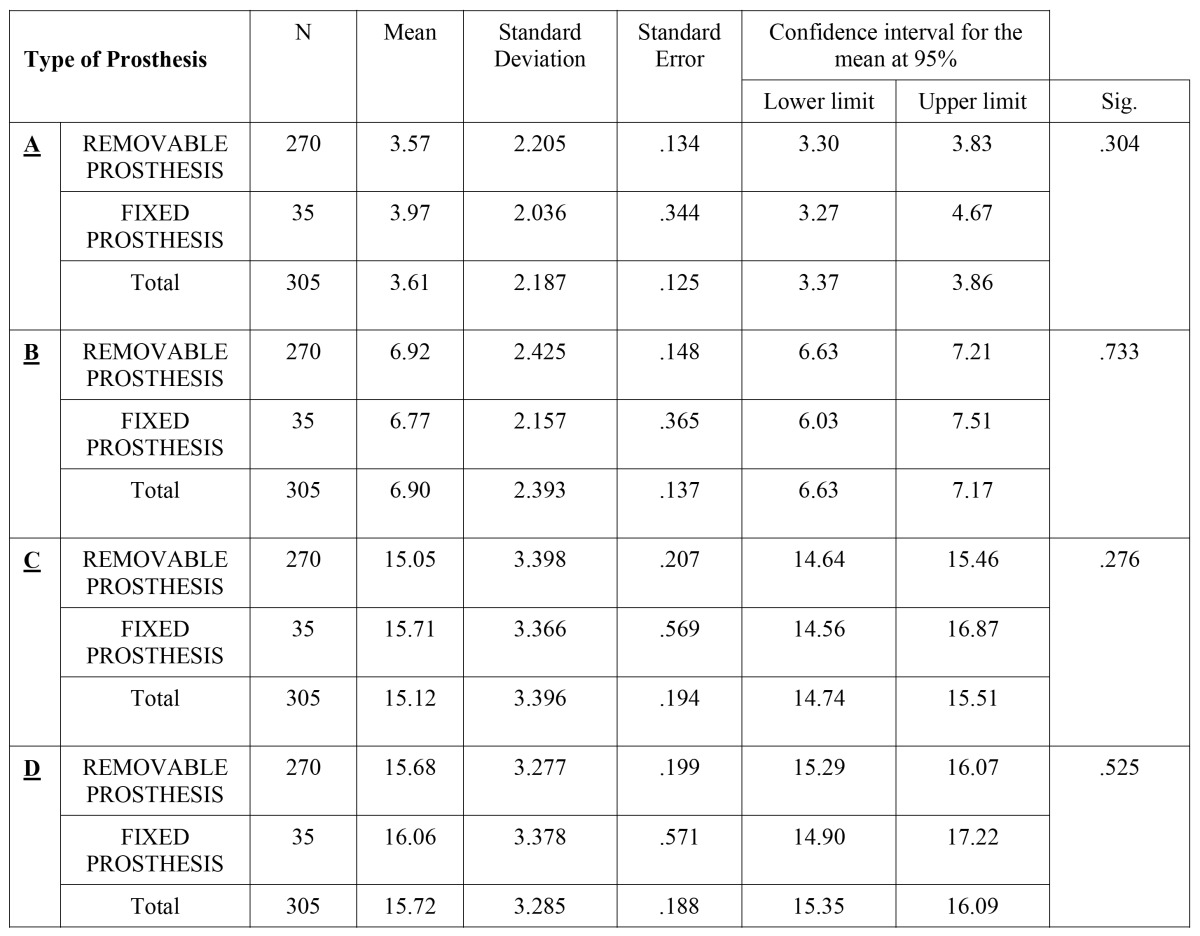
Descriptive and analytical analysis of the variable “Type of Prosthesis” (ANOVA Test).

## Discussion

The extraction of a tooth, due to cessation of asorption of forces from chewing initiates a degenerative process that involves a process of resorption of the alveolar bone, which is most significant in the first three months and decreases after 6 months, stabilizing within the first or second year following extraction (7,8). The rate of bone resorption, due to the influence of a series of local and systemic factors, varies between two individuals and even in a same individual at different times (3). In this study, among the factors that have been related with the asorption of the alveolar ridge, we have selected the sex of the patient, the type of prosthesis and antagonist. 

The first variable analyzed was the patients’ sex, observing that both the width and the height are significantly less in women versus in men. Along this same line of results, Xie et al. (2), in a study on 177 edentulous patients, concluded that women present a high risk of severe bone resorption. In addition, De Baat et al. (5), in a study on 175 patients, observed a different degree of bone resorption, noting it to be higher in women versus in men. Authors such as Bras and Bays (4,9) have determined that the smaller size of the ridge could be due to the effects of the deficiency of estrogens following menopause. Thus, studies conducted by Kribbs (10) and Klemetti et al. (11) show a relationship between alveolar atrophy and osteoporosis, which may be explained by a decrease in bone mass and bone mineral density at the level of the maxillae. In a study carried out by Kribbs (12), he analyzed whether osteoporosis, characterized by a decrease in bone mineral density (BMD), is a risk factor in bone resorption at the level of the maxillae, concluding that symptomatic osteoporosis could be a risk factor for a smaller-size residual alveolar ridge at the level of the maxillae, whereas it does not appear to be a risk factor at the mandibular level.

The next variable analyzed was the type of prosthesis, initially establishing three categories, but due to an insufficient sample size of the category “No Prosthesis” (NP), we only compared “Removable Dental Prosthesis” (RP) with “Fixed Dental Prosthesis”, observing that except in the width at the center, the lowest values correspond to RP, although the differences are not statistically significant (p>0.05). In this sense, Xie et al. (13) conclude that wearing a removable dental prosthesis could be a risk factor associated with a higher percentage of bone resorption following dental extraction. In addition, studies conducted by Xie et al. (13) and De Baat et al. (5) show a higher percentage of bone resorption when there is a poor fit of the prostheses and when patients have worn the prosthesis during the day and at night.

There are experimental studies in the literature, such as those conducted by Imao et al. and Sato et al. (14,15), in which bone resorption was observed to be induced by the continuous pressure exerted on the tissues by those who wear removable dental prostheses. In this study, we analyzed whether the size of the residual ridge varies according to the mechanical stress exerted by the antagonist on the tissues by wearers of removable dental prostheses. In our results, we observed that both in height as well as in width, the smaller size of the ridge corresponds to FP (Fig. 2), which is that which would exert greater pressure, although these differences are statistically significant only in width (p<0.01). In the literature, there is a lot of variability in the methodology and in the results. In the same manner, De Baat et al. (5) observed greater resorption at the level of the premaxilla in patients that present anteroinferior teeth, compared to patients who wear a full dental prosthesis or who have front and back teeth. In contrast, in the study carried out by Xie et al. (13), they did not find any significant differences. On the other hand, Jacobs et al. (16) observed greater bone loss in the group of patients with a full mandibular prosthesis, compared to those who wore a fixed dental prosthesis or an implant-supported denture. 
